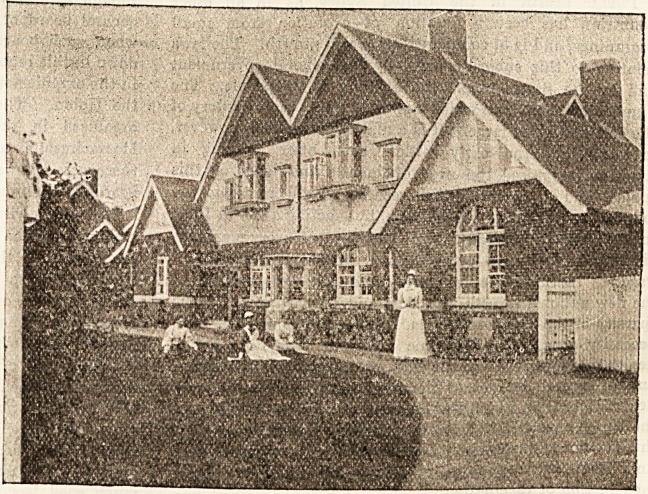# The Epileptic Colony at Chalfont St. Peter

**Published:** 1906-01-06

**Authors:** 


### HOSPITAL ADMINISTRATION.

### CONSTRUCTION AND ECONOMICS.

### THE EPILEPTIC COLONY AT CHALFONT ST. PETER.

THE Homes, each a moderately-sized, prettily built house, with accommodation for 18 to 24 colonists, are situated on a farm at Chalfont St. Peter, known as "The Colony." The land belonging to the colony consists of 210 acres, and includes a very large fruit-garden. Several homes for men and three for women are now open. The new administrative building contains a central kitchen, matron's rooms and office, and accommodation for the nursing staff. Two wings and a water tower will be added so soon as funds are forthcoming for them. Each home is in the charge of a sister, who has a nurse (in the case of the men's homes it is a male attendant) under her. The colonists sleep in dormitories, each containing about 9 to 12 beds; and an attendant sleeps in an adjoining cubicle, overlooking the dormitories, in order to be at hand to give assistance in the event of a colonist having a fit during the night. In each of the homes there is a sick-room for colonists suffering from any temporary ailment.

The names of some of the houses are the "Eleanor," the "Pearman," the "Milton," the "Greene," the "Hampshire," and the "Victoria." A different architect was responsible for each house because the Committee have always been anxious to prevent monotony in the buildings. One of their chief aims is to do away with any institutional feeling, and certainly these prettily built and well appointed detached houses, each standing in its own grounds, are conducive to the desirable atmosphere of home life. The sister, nurse, and inmates of each firmly believe their own house to be the best of all. But it must be admitted that the average visitor will pronounce the "Eleanor" to be the most beautiful. It has, for one thing, a very handsome hall, with polished wood floor, and walls adorned by a beautiful frieze, painted and presented by the Kyrle Society. The "Pearman" and "Milton" homes possess two lime-trees planted by the hands of the Prince and Princess of Wales.

There is a convalescent home, intended for epileptics convalescing from illness or accident, and also for the admission of holiday cases and others where a temporary stay at the colony is likely to be of benefit.

In addition to the arrangements for grown-up people, there is also accommodation for boys above the age of about 14, and girls above the age of about 16. Homes for young children from the age of 7 upwards will be built as soon as opportunity offers.

The Committee are most anxious to provide, as far as possible, for every colonist, of whatever denomination, the means of enjoying the privileges and comforts of religion. Church of England and Nonconformist services are as a rule held at the colony weekly, and arrangements are also made to enable colonists to go occasionally to services in the adjoining villages. Attendance at any of the services is entirely voluntary so far as colonists of full age are concerned. The health of the colonists is as a rule excellent, and since August 1897 there has been only one death. No patient is discharged as cured unless he has been free from fits for over two years. Patients are often removed by their friends under the belief that they are sufficiently recovered to be able to resume their ordinary avocations, though the honorary medical staff would consider it wiser to leave them at the colony for a somewhat longer period in order to confirm and establish the cure.

With the extension of the colony the methods at the disposal of the Committee for interesting and employing the inmates have been increased. In addition to the original forms of work, such as gardening, farm-work, and carpentering, there have also been added plumbing, painting, bricklaying, and smith's work, also basket-mending, tailoring, and boot-mending. It has not been found that in suitably chosen instances the more sedentary forms of work are detrimental to the health of the workers. On the female side the chief forms of work are confined to the house, the laundry, and needlework. The selection for these varieties of work depends chiefly upon the physical health of the colonist on admission, but few women have been found unable to undertake the more onerous duties of the laundry some weeks after arrival. The usual working time is about seven hours daily, except during the short days of winter, when it is somewhat less.

In visiting the settlement one is struck by the high standard of the work accomplished by the sufferers. At the Home Arts and Industries Exhibition the colony has been very successfully represented by a variety of work done both by the men and women; and at the opening of the last exhibition by the Queen, her Majesty inspected the Chalfont exhibits, and purchased some very beautiful work executed by one of the women. Another of the women colonists gained for her needlework the gold cross, which is the highest mark awarded at this exhibition.

For recreation, both indoor and outdoor games and amusements are encouraged and provided. And in their cricket and football matches against teams from the neighbouring towns and villages, the colonists have done remarkably well.

With regard to the eligibility and ineligibility of the cases, the colony, as at present constituted, is intended only for those epileptics whose mental and moral condition would enable them to become suitable members of such a community. Excessive frequency or severity of the fits does not disqualify, provided that the condition of the patient is in other respects satisfactory. There will ultimately be accommodation for three classes of patients. At present only third-class patients can be received, and the payment required is at the rate of ten shillings a week. In exceptional cases this sum is reduced, and Poor-law guardians have power to make the necessary payments for the maintenance of patients at the colony.

It is earnestly to be hoped that funds for the extension of the colony may be forthcoming, since the number of applicants is large in proportion to the available accommodation. The removal of an epileptic from unsuitable surroundings is a most humane action. Many thousands of families which might otherwise be happy and prosperous are reduced to misery and despair by the presence of an epileptic in their midst. And the life of the epileptic himself (or herself) is often one of involuntary idleness, which leads to mental and moral deterioration, and to the serious aggravation of the disease. The more capable and industrious they are by nature, the more depressing and demoralising, in the long run, are the effects of their aimless and empty existences. Some compensation is due to them, especially when it is realised that, apart from the epilepsy, they are not inferior to their fellows; indeed, several of the greatest men in history, including Julius Cæsar, Mahomet, and Napoleon, are reputed to have been epileptics. And the very high attainments of many of the colonists at Chalfont St. Peter is but another proof of the saying that "the invalids do the work of the world."

## Figures and Tables

**Figure f1:**
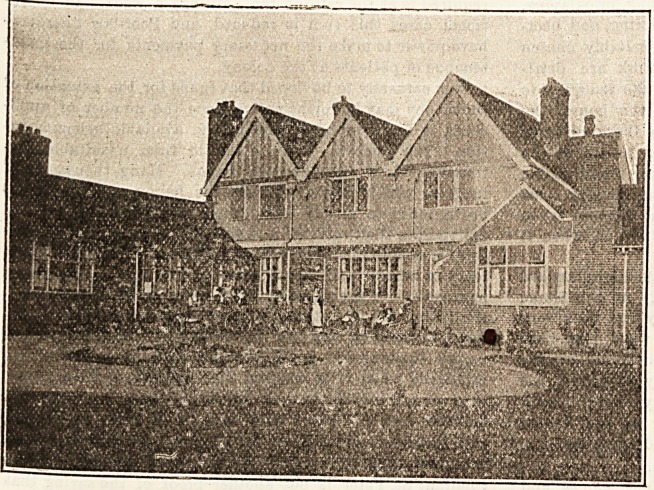


**Figure f2:**